# Supplementary material for: Bridging the TB data gap: in silico extraction of rifampicin-resistant tuberculosis diagnostic test results from whole genome sequence data
Source: PeerJ. 2019 Aug 26;7:e7564. doi: 10.7717/peerj.7564 (PMC6714962; doi:10.7717/peerj.7564)
Supplement: Supplemental Information 1 [file peerj-07-7564-s001.pdf]

Supplemental File

Table S1. Summary tables of counts and proportions of rifampicin-susceptible TB samples, rapid diagnostic test probes, and rifampicin-resistant TB mutations detected by the TBGT tool from the 2005 to 2010 isolates in Kinshasa, DRC.

Xpert classic probe	counts
Rif Sensitive	59
ProbeB	27
ProbeB,ProbeE_delayed	3
ProbeB_delayed	8
ProbeC	12
ProbeD	20
ProbeD,ProbeE_delayed	1
ProbeD_delayed	5
ProbeE	185
ProbeE_delayed	4

Xpert classic probe	proportions
Rif Sensitive	0.182
ProbeB	0.083
ProbeB,ProbeE_delayed	0.009
ProbeB_delayed	0.025
ProbeC	0.037
ProbeD	0.062
ProbeD,ProbeE_delayed	0.003
ProbeD_delayed	0.015
ProbeE	0.571
ProbeE_delayed	0.012

Xpert Ultra probe	counts
Rif Sensitive	59
rpoB1,rpoB2	4
rpoB2	31
rpoB2,rpoB3	12
rpoB2,rpoB4B	3
rpoB3	25
rpoB3,rpoB4A	185
rpoB3,rpoB4B	1
rpoB4B	4

Xpert Ultra probe	proportions
Rif Sensitive	0.182
rpoB1,rpoB2	0.012

rpoB2	0.096
rpoB2,rpoB3	0.037
rpoB2,rpoB4B	0.009
rpoB3	0.077
rpoB3,rpoB4A	0.571
rpoB3,rpoB4B	0.003
rpoB4B	0.012

Hain LPA probe	counts
Rif Sensitive	59
WT2,WT3,WT4,MUT1	4
WT3,WT4	9
WT3,WT4,MUT1	22
WT3,WT8	3
WT5,WT6	12
WT7	11
WT7,MUT2A	7
WT7,MUT2B	7
WT7,WT8	1
WT8	5
WT8,MUT3	184

Hain LPA probe	proportions
Rif Sensitive	0.182
WT2,WT3,WT4,MUT1	0.012
WT3,WT4	0.028
WT3,WT4,MUT1	0.068
WT3,WT8	0.009
WT5,WT6	0.037
WT7	0.034
WT7,MUT2A	0.022
WT7,MUT2B	0.022
WT7,WT8	0.003
WT8	0.015
WT8,MUT3	0.568

Nipro LPA probe	counts
Rif Sensitive	59
S1,S2,R2	4
S2	9
S2,R2	22
S2,S5	3
S3	12
S4	11
S4,R4a	7

S4,R4b	7
S4,S5	1
S5	5
S5,R5	184

Nipro LPA probe	proportions
Rif Sensitive	0.182
S1,S2,R2	0.012
S2	0.028
S2,R2	0.068
S2,S5	0.009
S3	0.037
S4	0.034
S4,R4a	0.022
S4,R4b	0.022
S4,S5	0.003
S5	0.015
S5,R5	0.568

Sanger probe	counts
Asp435Phe	1
Asp435Tyr	7
Asp435Val	22
Gln432Glu,Asp435Val	4
His445Arg	5
His445Asn,Leu452Pro	1
His445Asp	7
His445Leu	5
His445Thr	1
His445Tyr	7
Ile491Phe	5
Leu430Arg,Asp435Gly	1
Leu452Pro	4
Met434Ile,Leu452Pro	3
Rif Sensitive	54
Ser441Gln	12
Ser450Leu	184
Ser450Trp	1

Sanger probe	proportions
Asp435Phe	0.003
Asp435Tyr	0.022
Asp435Val	0.068
Gln432Glu,Asp435Val	0.012
His445Arg	0.015

His445Asn,Leu452Pro	0.003
His445Asp	0.022
His445Leu	0.015
His445Thr	0.003
His445Tyr	0.022
Ile491Phe	0.015
Leu430Arg,Asp435Gly	0.003
Leu452Pro	0.012
Met434Ile,Leu452Pro	0.009
Rif Sensitive	0.167
Ser441Gln	0.037
Ser450Leu	0.568
Ser450Trp	0.003
